# Hospital admissions in the first year of life: inequalities over three decades in a southern Brazilian city

**DOI:** 10.1093/ije/dyy228

**Published:** 2019-03-18

**Authors:** Fernando C Wehrmeister, Cesar G Victora, Bernardo L Horta, Ana M B Menezes, Iná S Santos, Andréa Dâmaso Bertoldi, Bruna G C da Silva, Fernando C Barros, Pelotas Cohorts Study Group, Aluisio J D Barros, Alicia Matijasevich, Diego G Bassani, Helen Gonçalves, Joseph Murray, Luciana Tovo-Rodrigues, Maria Cecilia F Assunção, Mariangela F Silveira, Marlos Rodrigues Domingues, Pedro R C Hallal

**Affiliations:** 1 Federal University of Pelotas, Brazil; 2 University of São Paulo, Brazil; 3 University of Toronto, Canada; 1 Postgraduate Program in Epidemiology, Federal University of Pelotas, Pelotas, Brazil; 2 Postgraduate Program in Health and Behavior, Catholic University of Pelotas, Pelotas, Brazil

**Keywords:** Child health, hospitalization, health care disparities, socioeconomic factors, cohort studies

## Abstract

**Background:**

Hospital admissions in infancy are declining in several countries. We describe admissions to neonatal intensive care units (NICU) and other hospitalizations over a 33-year period in the Brazilian city of Pelotas.

**Methods:**

We analysed data from four population-based birth cohorts launched in 1982, 1993, 2004 and 2015, each including all hospital births in the calendar year. NICU and other hospital admissions during infancy were reported by the mothers in the perinatal interview and at the 12-month visit, respectively. We describe these outcomes by sex of the child, family income and maternal skin colour.

**Results:**

In 1982, NICUs did not exist in the city; admissions into NICUs increased from 2.7% of all newborns in 1993 to 6.7% in 2015, and admission rates were similar in all income groups. Hospitalizations during the first year of life fell by 29%, from 23.7% in 1982 to 16.8% in 2015, and diarrhoea admissions fell by 95.2%. Pneumonia admissions fell by 46.3% from 1993 to 2015 (no data available for 1982). Admissions due to perinatal causes increased during the period. In the poorest income quintile, total admissions fell by 33% (from 35.7% to 23.9%), but in the richest quintile these remained stable at around 10%, leading to a reduction in inequalities. Over the whole period, children born to women with black or brown skin were 30% more likely to be admitted than those of white-skinned mothers.

**Conclusions:**

Whereas NICU admissions increased, total admissions in the first year of life declined by nearly one-third. Socioeconomic disparities were reduced, but important gaps remain.

**Key Messages**

- Hospital admissions in the first year of life fell from 23% of all infants in 1982 to 16.8% in 2015.
- The decline was particularly steep for diarrhoea admissions, which fell from being the leading cause of admissions in 1982 to virtual disappearance by 2015.
- Admissions due to complications of prematurity increased over time, as measured by NICU admissions (from 2.7% of all newborns in 1993 to 6.7% in 2015), as did admissions during the first year of life due to perinatal causes.
- NICU admissions were not related to family income or maternal skin colour.
- Income-related inequalities in admissions were reduced over time, but remained important up to 2015 with a 2-fold excess in the poorest compared with the richest quintiles.
- Over the whole period, children born to Black or Brown mothers were 30% more likely to be hospitalized in infancy than children of White mothers.

## Introduction

Hospital admissions are an important public health issue worldwide, with high costs for health systems, individuals and society. Infants and the elderly are the age groups at highest risk of hospitalization. In high-income countries, the risk of admission during the first year currently ranges widely, from 4.4%^1^ in New York to 31% in Australia.^2^ Some studies suggest declining trends in hospital admissions for infants, as was the case in New South Wales^3^ where the rate decreased by 1.8% per year from 2001 to 2009.

In Brazil, data from the National Information System indicates that hospitalization in the first year of life decreased from 24.4 in 1998 to 19.0 in 2015 per 100 live births.^4^ In 1998, the major causes of hospitalizations were related to respiratory disease (34%), followed by perinatal conditions (14%) and other infectious diseases (22%), whereas in 2017 this picture changed: 46% of hospital admission were due to perinatal conditions, 24% due to respiratory diseases and only 11% due to other infectious diseases.^4^

Regarding inequalities in hospital admission, children living in urban settings are at high risk of hospitalization compared with those who live in rural areas.^5^ Also, socially deprived ethnic groups^1^^,^^6^^,^^7^ are at greater risk of hospital admission, the same occurring with emergency hospital admissions.^8^

Time trends in hospital admissions in the Pelotas (Brazil) 1982, 1993 and 2004 birth cohorts were reported in 2008.^9^ In this report, we extend this time series to also include the 2015 cohort, covering a period of three decades, and assess admissions to neonatal intensive care units (NICU). Our analyses report levels and trends in inequalities associated with family income, child sex and maternal skin colour.

## Methods

All live births occurring in the city of Pelotas, Brazil, during the calendar years of 1982, 1993, 2004 and 2015 were included in each cohort study. From 1 January to 31 December 31 each year, all urban women delivering in the city’s hospitals were invited to participate. The four cohorts included 5914, 5249, 4231 and 4275 live births, respectively.^10^

NICU admissions were based on maternal report at the perinatal interview that took place soon after delivery. All infants referred to an NICU were included in the analysis, irrespective of the duration of hospitalization. This information was not collected in 1982, when NICUs were not yet available in the city.

In the first cohort, information on hospital admissions was collected in early 1983 when children born from January to April 1982 were sought at home, and their mothers interviewed. Because the most frequent cause for admission was diarrhoea, information on cause was only collected for this condition, and all other causes were grouped together. A validation study comparing the responses of 120 mothers with hospital charts showed that the underlying cause of hospitalization had been correctly provided by the mother in 90% of cases.^11^

For the 1993 birth cohort, all babies born weighing less than 2500 g, and a random sample of 20% of all other children, were sought at the age of 12 months. On this occasion, mothers were asked about hospitalizations and their causes. In addition, a detailed study of hospital admissions was conducted in this cohort. The four city hospitals with paediatric wards were visited on a weekly basis during 1993 and 1994 to identify admissions of cohort members. Two independent reviewers analysed the available information and determined the cause of hospitalization according to the ninth International Classification of Diseases (ICD-9). In case of disagreement, a senior referee took the final decision. Information was available for over 98% of hospitalizations of infants during this period.^12^ Weighted data analyses were carried out to account for the oversampling of low-birthweight infants in 1993.

In 2004 and 2015, an attempt was made to locate all cohort members at the age of 12 months. Information on hospitalizations and causes was collected from the mothers, and the variables were recorded in a similar way as in the 1993 birth cohort.

Hospitalizations were defined as inpatient admissions lasting more than 24 h. In all cohorts, all-cause and diarrhoea admissions were recorded. From 1993 to 2015, information was also recorded on pneumonia admissions and on perinatal causes. The latter were recorded when a perinatal condition was reported (e.g. prematurity or neonatal sepsis), or when the baby was hospitalized during the first month of life due to causes other than diarrhoea or pneumonia. Among all children who were hospitalized in the four cohorts, 26% were admitted more than once. Therefore, the total number of children admitted to hospital does not correspond to the sum of the groups of causes. All analyses were repeated considering only the first admission per child.

The frequencies of NICU and hospital admissions were analysed according to child sex (male/female), family income (number of monthly minimum wages received by the family, divided in quintiles) and maternal skin colour (white, brown and black, observed by the interviewer in 1982 and 1993 and self-reported in 2004 and 2015). For family income, we also calculated the slope index of inequality (SII) for absolute inequality and the concentration index (CIX) for relative inequality. The SII is a measure based on the difference in the values of a given outcome between the extremes of the distribution, through a logistic regression for binary outcomes.^13^ It is expressed in percentage points ranging from −100 and 100, with the zero representing no inequality, and negative values are translated as the poorest group having high prevalence of the outcome. The CIX is a relative inequality measure similar measure to the Gini index, and uses the concentration of a given outcome according to socioeconomic status.^13^ It is also expressed in a range from −100 to 100, however with no defined unit. The interpretation is similar to the SII. For sex and maternal skin colour, we calculated the absolute (arithmetical difference) and relative (ratio) inequalities for male/female and Black/White, respectively.

We used the statistical software Stata, version 13.^14^ We calculated the bivariate association in each cohort using the chi square test for heterogeneity for the variables sex and skin colour, and for linear trend in the case of income quintiles. To analyse changes over time, we used the chi square test for trend. Interactions between exposures and cohort year were tested through Poisson regression. In the presence of interaction, we presented the results separately for each cohort. In the absence of interaction, pooled results were presented.

The protocols of each phase in each cohort were approved by the Institutional Review Board of the Federal University of Pelotas. In the years 1982 and 1993, a verbal consent from the mothers was obtained. In 2004 and 2015, a consent form was filled and signed by the mothers who were fully informed and accepted participation in the study.

## Results

The numbers of children in the analyses were 1462 in 1982 (subsample), 1363 in 1993 (subsample), 3907 in 2004 and 4015 in 2015, corresponding to response rates of 79.5%, 93.4%, 94.4% and 95.4%, respectively, of eligible children.

NICU admission increased from 2.7% in 1993 to 6.7% in 2015. The frequency of hospitalizations due to any cause decreased from 23.7% in 1982 to 18.1% in 1993, and remained nearly stable until 2015, when it reached 16.8% (Table 1). Important reductions in hospital admissions due to diarrhoea and pneumonia were observed over time. The prevalence of hospitalizations due to perinatal causes (not available in 1982) was 2.0% in 1993, 7.0% in 1993 and 5.1% in 2015. The mean number of hospitalizations per child ranged from zero to six. The average number decreased from 0.4 [standard deviation (SD) = 0.8] to 0.2 (SD = 0.7) over the three decades (Table 1).

**Table 1.** Percentages of cohort children admitted to NICUs and of hospital admissions in the first year of life, according to cause

Admission and cause	Birth cohort				*P*-value*
	1982	1993	2004	2015	
NICU admission	0	2.7 (1.9 to 3.4)	5.1 (4.5 to 5.8)	6.7 (5.9 to 7.5)	<0.001
Diarrhoea	6.3 (5.0 to 7.5)	2.4 (1.5 to 3.3)	1.0 (0.7 to 1.3)	0.3 (0.2 to 0.5)	<0.001
Pneumonia	–	6.5 (5.1 to 7.8)	4.4 (3.8 to 5.1)	3.5 (2.9 to 4.1)	<0.001
Perinatal causes	–	2.0 (1.2 to 2.8)	7.0 (6.2 to 7.8)	5.1 (4.4 to 5.7)	0.015
Other causes	–	9.3 (7.8 to 11.0)	9.4 (8.6 to 10.4)	10.2 (9.4 to 11.3)	0.196
Any cause^a^	23.7 (21.6 to 25.9)	18.1 (16.1 to 20.1)	19.2 (17.9 to 20.4)	16.8 (15.7 to 18.0)	<0.001
Mean number of hospitalizations/child	0.4 (0.8)	0.3 (0.8)	0.3 (0.6)	0.2 (0.7)	<0.001

a The total number of children admitted to hospital does not correspond to the sum of the groups of causes, because some children may have been admitted more than once due to different causes.

* *P*-value for chi square test for linear trend in the four cohorts.

Table 2 shows NICU admissions in the three more recent birth cohorts, analysed by the child’s sex, family income quintiles and maternal skin colour. Admissions increased over time in all subgroups and was slightly higher for male babies compared with females, especially in 2015. Regarding income groups, there was little statistical evidence of inequality in any of the cohorts, with the 95% confidence intervals for the slope and concentration indices including the null value. There was no interaction (*p* = 0.544) between family income and cohort in NICU admissions. In relation to maternal skin colour, babies born to Black mothers were more likely to be admitted than those with White mothers in 1993, but there was no evidence of a difference in 2015 (*p*-value for interaction = 0.010).

**Table 2.** Percentages of cohort children admitted to NICU according to sex, family income and maternal skin colour

Variable	Birth cohort year				*P*-value**
	1982	1993	2004	2015	
	% (95% CI)	% (95% CI)	% (95% CI)	% (95% CI)	
Sex		*P* = 0.927*	*P* = 0.116*	*P* = 0.041*	
Male	–	2.6 (1.5; 3.7)	5.7 (4.7; 6.7)	7.5 (6.4; 8.6)	<0.001
Female	–	2.7 (1.7; 3.7)	4.6 (3.6; 5.5)	5.9 (4.8; 6.9)	<0.001
Difference male–female	–	−0.1	1.1	1.6	
Ratio male/female	–	1.0	1.2	1.3	
Family income (quintiles)		*P* = 0.221*	*P* = 0.216*	*P* = 0.532*	
Q1 (poorest)	–	2.5 (1.0; 4.0)	6.5 (4.8; 8.3)	7.6 (5.7; 9.5)	0.002
Q2	–	3.7 (1.8; 5.6)	5.0 (3.5; 6.6)	6.7 (5.0; 8.5)	0.025
Q3	–	2.1 (0.5; 3.7)	3.9 (2.5; 5.3)	5.9 (4.3; 7.6)	0.003
Q4	–	2.3 (0.7; 3.9)	5.5 (4.0; 7.1)	6.3 (4.7; 8.0)	0.013
Q5 (richest)	–	1.8 (0.5; 3.1)	4.7 (3.2; 6.2)	6.9 (5.1; 8.7)	<0.001
Slope index of inequality	–	−0.7 (−5.0; 3.5)	−1.6 (−4.1; 0.9)	−0.9 (−3.6; 1.9)	
Concentration index	–	−1.7 (−14.8; 11.4)	−4.2 (−12.2; 3.8)	−1.9 (−8.8; 5.0)	
Maternal skin colour		*P* = 0.003*	*P* = 0.752*	*P* = 0.196*	
White	–	2.1 (1.4; 2.8)	5.1 (4.3; 5.9)	7.0 (6.1; 7.9)	<0.001
Brown	–	3.8 (0.0; 8.5)	4.8 (2.2; 7.3)	6.3 (4.2; 8.4)	0.280
Black	–	5.1 (2.5; 7.8)	5.4 (3.8; 7.0)	5.6 (3.8; 7.5)	0.768
Difference Black-White	–	3.0	0.3	−1.4	
Ratio Black/White	–	2.4	1.1	0.8	

* *P*-values for differences within each cohort.

** *P*-values for time trends within each category.

The frequency of hospitalization for all causes is presented in Table 3. Important socioeconomic differences were observed in all four cohorts. There was no evidence of an interaction between sex and cohort year (*p* = 0.214), so that the results from the four cohorts were pooled. Overall, boys were 23% [95% confidence intervalCI) 1.13 to 1.33] more likely than girls to be admitted in all four cohort. In 1982, 35% of the poorest babies were hospitalized compared with 8.2% in the richest quintile. Over the three decades, admissions in the poorest quintile fell from 35.7% to 23.9%, whereas no changes over time were observed in the two richest quintiles. There was evidence of an interaction between income and cohort year (*p*  = 0.020). The SII was reduced from -33.0% points in 1982 to −15.9 in 2015, and the CIX from −23.9 to −15.4. For maternal skin colour, there was no evidence of interaction (*p* = 0.299) and the pooled prevalence ratio was 1.26 (95% CI 1.16 to 1.38).

**Table 3.** Percentages of cohort children admitted to a hospital due to any cause during the first year of life, according to sex, family income and maternal skin colour

Variable	Birth cohort year				*P*-value**
	1982	1993	2004	2015	
	% (95% CI)	% (95% CI)	% (95% CI)	% (95% CI)	
Sex	*P* = 0.031*	*P* = 0.142*	*P* = 0.208*	*P*<0.001*	
Male	26.2 (23.0 to 29.4)	19.6 (16.7 to 22.5)	19.9 (18.2 to 21.7)	19.7 (18.0 to 21.4)	0.005
Female	21.4 (18.4 to 24.3)	16.7 (14.0 to 19.4)	18.3 (16.6 to 20.1)	13.8 (12.3 to 15.4)	<0.001
Difference male -female	4.8	3.2	1.6	5.9	
Ratio male/female	1.2	1.2	1.1	1.4	
Family income (quintiles)	*P*<0.001*	*P*<0.001*	*P*<0.001*	*P*<0.001*	
Q1 (poorest)	35.7 (29.4 to 42.0)	24.2 (18.7 to 29.7)	24.8 (21.7 to 27.8)	23.9 (20.9 to 26.8)	0.004
Q2	30.9 (25.8 to 36.1)	20.0 (15.3 to 24.6)	23.4 (20.4 to 26.4)	20.1 (17.3 to 22.8)	0.002
Q3	29.3 (24.3 to 34.4)	15.4 (10.8 to 20.0)	19.6 (16.8 to 22.4)	15.7 (13.2 to 18.3)	<0.001
Q4	17.5 (13.2 to 21.7)	15.9 (11.1 to 20.6)	15.4 (12.9 to 17.9)	13.8 (11.4 to 16.2)	0.113
Q5 (richest)	8.2 (5.1 to 11.3)	8.0 (4.6 to 11.3)	12.7 (10.4 to 15.1)	10.8 (8.6 to 12.9)	0.107
Slope index of inequality	−33.0 (−39.8 to −26.1)	−13.8 (−21.2 to −6.3)	−15.7 (−19.9 to −11.5)	−15.9 (−19.9 to −11.9)	
Concentration index	−23.9 (−28.6 to −19.2)	−10.0 (−15.9 to −4.1)	−13.6 (−17.3 to −9.9)	−15.4 (−19.3 to −11.5)	
Maternal skin colour	*P* = 0.024*	*P* = 0.297*	*P* = 0.045*	*P*<0.001*	
White	22.5 (20.2 to 24.9)	16.7 (14.3 to 19.1)	18.3 (16.9 to 19.8)	15.1 (13.8 to 16.4)	<0.001
Brown	29.0 (23.4 to 34.5)	19.2 (9.5 to 29.0)	21.8 (16.8 to 26.7)	20.3 (16.8 to 23.7)	0.075
Black		19.5 (14.2 to 24.8)	21.2 (18.3 to 24.1)	22.0 (18.7 to 25.3)	
Difference Black-White	6.5	2.8	2.9	6.9	
Ratio Black/White	1.3	1.2	1.2	1.5	

* *P*-values for differences within each cohort.

** *P*-values for time trends within each category.

In Table 4, results for hospital admissions due to diarrhoea are presented. Within any cohort, there were no differences by sex of the child. From 1982 to 2015, admissions were reduced from 6.7% to 0.4% in males and from 5.9% to 0.3% in females. Except for 2015 when diarrhoea admissions were extremely rare, the frequencies were much higher among children in the poorest quintile than in the richest quintile. Absolute inequalities fell from -11.7% points in 1982 to -0.1 in 2015, with confidence intervals that did not overlap. Relative inequalities remained stable from 1982 to 2004, with a marked drop in 2015. Possibly because of very small numbers in 2015, the Poisson regression test for interaction between income and cohort year had a large *p*-value of 0.820; this test refers to relative inequalities, given the multiplicative nature of Poisson regression. Regarding skin colour, there was no interaction with cohort year (*p* = 0.562) and the pooled analysis of the four cohorts showed a 40% excess (95% CI 1.00 to 1.95) of diarrhoea admissions for children born to Black or Brown mothers, compared with those with White mothers.

**Table 4.** Percentages of cohort children admitted to a hospital due to diarrhoea during the first year of life, according to sex, family income and maternal skin colour

Variable	Birth cohort year				*P*-value**
	1982	1993	2004	2015	
	% (95% CI)	% (95% CI)	% (95% CI)	% (95% CI)	
Sex	*P* = 0.544*	*P* = 0.944*	*P* = 0.564*	*P* = 0.637*	
Male	6.7 (4.9 to 8.5)	2.4 (1.1 to 3.6)	1.1 (0.6 to 1.5)	0.4 (0.1 to 0.7)	<0.001
Female	5.9 (4.2 to 7.6)	2.4 (1.2 to 3.6)	0.9 (0.5 to 1.3)	0.3 (0.0 to 0.5)	<0.001
Difference male -female	0.8	0.0	0.1	0.1	
Ratio male/female	1.1	1.0	1.1	1.3	
Family income (quintiles)	*P*<0.001*	*P* = 0.001*	*P*<0.001*	*P* = 0.615*	
Q1 (poorest)	12.1 (7.8 to 16.3)	6.0 (2.8 to 9.0)	2.3 (1.3 to 3.4)	0.1 (0.0 to 0.4)	<0.001
Q2	7.2 (4.3 to 10.1)	2.7 (0.8 to 4.5)	1.0 (0.3 to 1.7)	0.7 (0.2 to 1.3)	<0.001
Q3	8.5 (5.4 to 11.6)	1.2 (0.0 to 2.6)	0.9 (0.2 to 1.6)	0.2 (0.0 to 0.6)	<0.001
Q4	3.6 (1.5 to 5.6)	1.4 (0.0 to 2.9)	0.4 (0.0 to 0.8)	0.5 (0.0 to 1.0)	<0.001
Q5 (richest)	1.6 (0.2 to 3.1)	0.6 (0.0 to 1.6)	0.4 (0.0 to 0.8)	0.1 (0.0 to 0.4)	0.005
Slope index of inequality	−11.7 (−16.2 to −7.1)	−5.2 (−8.7 to −1.6)	−2.3 (−3.5 to −1.0)	−0.1 (−0.6 to 0.4)	
Concentration index	−28.5 (−38.5 to −18.5)	−30.9 (−48.7 to −13.1)	−36.5 (−53.2 to −19.8)	−2.7 (−23.1 to 17.7)	
Maternal skin colour	*P* = 0.447*	*P* = 0.026*	*P* = 0.447*	*P* = 0.525*	
White	6.1 (4.7 to 7.4)	1.8 (1.0 to 2.6)	0.9 (0.6 to 1.3)	0.3 (0.1 to 0.6)	<0.001
Brown	7.3 (4.2 to 10.5)	6.1 (0.0 to 12.3)	0.7 (0.0 to 1.8)	0.0	<0.001
Black		4.1 (1.2 to 6.9)	1.3 (0.5 to 2.1)	0.7 (0.0 to 1.3)	
Difference Black-White	1.2	2.3	0.4	0.4	
Ratio Black/White	1.2	2.3	1.4	2.3	

* *P*-values for differences within each cohort.

** *P*-values for time trends within each category.

Analysis for hospital admissions due to perinatal causes (Supplementary Table S1, available as Supplementary data at *IJE* online) and pneumonia (Supplementary Table S2, available as Supplementary data at *IJE* online) are presented in the Supplementary materials, available as Supplementary data at *IJE* online. In general, the patterns of associations and trends are similar to those observed for diarrhoea.

All analyses were repeated including only the first admission for each child, and results were virtually unchanged (data not presented).

## Discussion

Our results show an increase in NICU use after birth from 1993 to 2015. This was likely related to the sharp increase in preterm deliveries during the period, from about 6% in 1982 to 15% in 2015,^15^ as well as to the increased availability of NICU beds in more recent years. In 2004 and 2015, neither socioeconomic nor ethnic inequalities in NICU admissions were evident, but boys were more likely to be admitted than girls. The largest increases over time were observed for babies born to White mothers (from 2.1 in 1993 to 7.0 in 2015) and to women in the highest income group (from 1.8 to 6.9). Possible reasons for these trends are discussed below.

The prevalence of hospitalizations in the first year of life decreased by about one-third. The decline was limited to the poorer quintiles, as admission rates were stable over time in the two richest quintiles. Despite the observed decrease in the gaps between socioeconomic groups, important inequalities still persist. Among causes for which data are available, the fastest decline was for diarrhoea, followed by pneumonia, whereas the relative share of perinatal causes increased markedly. Our results on hospital admissions are fully consistent—in terms of time trends, causes and socioeconomic disparities—with our analyses on infant mortality in the four cohorts, which are presented in an accompanying article in this issue.^16^ These analyses showed that infant mortality rates fell from 36.4 in 1982 to 13.8 per 1000 live births in 2015, with particularly rapid reductions in infectious causes.

A detailed study of which factors are likely responsible for the decline in admissions in Pelotas is beyond the scope of this analysis. An in-depth study of factors associated with the improved health and reduced mortality of Brazilian children since 1990^17^ shows the importance of changes in social determinants of health due to: poverty reduction (particularly the important increase in the value of the minimum wage and a massive programme of conditional cash transfers for poor families); improved access to health care with the creation of a national health system; vertical health programmes against infectious diseases (including diarrhoea, pneumonia and vaccine-preventable diseases); and improved breastfeeding practices, paralleled by a sharp reduction in undernutrition. Such national-level changes were also occurring in Pelotas. From 1982 to 2015, standards of housing, sanitation and water supply improved markedly, as well as levels of parental education and family income.^10^ Although low birthweight remained stable,^15^ undernutrition in infancy was markedly reduced.^18^ Parental smoking was also reduced.^19^ The prevalence of exclusive breastfeeding at 3 months, and of continued breastfeeding at 12 months, increased sharply.^20^ All of these changes must have contributed to reduced frequency of hospital admissions.

It is also important to account for changes in access to health care. In 1982, children from poor families were mostly admitted to a charity hospital ran by the Catholic Church. A common reason for admission for infectious diseases was the fact that poor families could not afford outpatient antibiotic treatment, and thus had to be admitted in order to receive such drugs at no out-of-pocket costs. In 1989, a universal national health system was created, which increased access for all citizens to inpatient and outpatient care. Access to antenatal, delivery and child health care also improved markedly after implementation of the national health service.^21^ In spite of increased access, however, hospital admission rates as well as mortality continued to decline, which suggests that children became healthier (which is consistent with the sharp reduction in undernutrition),^18^ and/or that increased access to outpatient care, including free antibiotics, reduced the need for hospital admission. Outpatient services were greatly expanded with the creation of the Family Health Strategy, a national programme launched in the 1990s^22^ which provided free care in low-income neighbourhoods. Both of the leading causes of hospitalization in 1982—diarrhoea and pneumonia—are highly sensitive to primary health care, and therefore the expansion of the Family Health Strategy is likely to have contributed to the decline in these causes.^23^

On the other hand, the marked increase in NICU admissions, in parallel with the growing prevalence of preterm births, has been related to poor quality of health care and to excessive medicalization of childbirth, including the fact that caesarean sections now account for over 60% of all deliveries in the city.^15^^,^^21^ Other articles in this supplement report on the increases in caesarean sections, in preterm and in early term deliveries, all of which may have contributed to the rise in NICU admissions^24^ by leading to iatrogenic premature births.^15^^,^^21^ It is noteworthy that the most rapid increases in admissions were observed for babies born to White and to high-income mothers—who are most likely to suffer excessive medicalization.

Our findings of a decline in all-cause hospitalizations, and particularly those due to diarrhoea and pneumonia, accompanied by an increase in perinatal causes, are similar to what is observed in Brazilian official data produced by the Unified Health System (SUS).^4^ From 1998 to 2015, all-cause admission rates decreased from 244 to 190 per 1000 live births. Similar trends were reported for hospitalizations due to respiratory diseases (from 85 to 52 per 1000 live births) and for other infectious diseases (from 56 to 23 per 1000 live births). The national data also reflect our observation of an increase in admissions due to perinatal conditions (from 59 to 80 per 1000 live births).^4^

Our analyses have the advantage of allowing disaggregation by socioeconomic position and skin colour, which is not possible with the national information system. Our findings on inequalities are consistent with earlier analyses from Pelotas,^11^ as well as with a study conducted in north-east Brazil in which children from low-income families were 2.3 times more likely to be admitted than children from better-off families.^25^

Our finding of inverse associations between child hospitalizations and socioeconomic position are also consistent with the literature from high-income countries like the USA,^26^^,^^27^ Canada^28^ and the UK.^2^^,^^6^^,^^29^^,^^30^ It appears that when economic barriers to hospitalization are small or non-existent, higher morbidity and severity of illnesses associated with poverty lead to higher admission rates among disadvantaged children compared with children from better-off families.

The literature on child hospitalizations according to socioeconomic position in low-income countries is very scarce, but it is likely that barriers to accessing hospital care may prevent children from poor families being admitted. Two studies from Tanzania report direct associations between socioeconomic position and child hospitalizations.^31^^,^^32^

We found that infants born to mothers with brown or black skin colour were about 30% more likely to be admitted during infancy than those born to white-skinned mothers, which is likely associated with family income.^10^ Again, these results are consistent with the ethnic group inequalities reported in high-income countries.^2^^,^^6^^,^^33^^,^^34^ It is possible that the lack of differences in hospitalizations according to skin colour observed in 2004 and 2015, as opposed to 1993, could be due to the reduced frequency of diarrhoea and pneumonia admissions among non-Whites, as well as to increased hospitalizations of preterm babies among better-off, predominantly White families.^15^

Our study has limitations. The first refers to ascertainment and classification of causes for admissions. In 1982, only diarrhea—the major cause for admission—and other causes were recorded at the 12-month interview. In 2004 and 2015, information collected at 12 months referred to several groups of causes. In 1993, a prospective sub-study collected detailed information on causes of admission based on interviews with the parents and review of hospital casenotes.^35^

It is unlikely, however, that these differences would have distorted the present findings, given the consistent time trends observed for the four cohorts. Information on NICU admissions was collected at the postnatal interview, when newborns were still in intensive care, and is unlikely to have been biased. Other limitations of our analyses—including the collection of information on skin colour and family income, and losses to follow-up—are discussed in the first article in this Supplement.^10^

Our findings regarding cause-specific time trends and inequalities associated with income and skin colour are consistent with the results on infant mortality.^16^ The reasons behind the reported improvements are multiple, including a reduction in poverty, increased parental educational levels, lower fertility and better housing, water supply and sanitation, as well as important improvements in breastfeeding practices and a reduction in maternal smoking.^10^^,^^16^^,^^19^^,^^21^^,^^36^ A more detailed discussion of the likely reasons behind the improvements in child health and nutrition in Brazil is available elsewhere.^17^ In spite of overall progress, important challenges remain such as the increase in admissions related to prematurity, and persistent social and ethnic group inequalities. Policies in the health and other sectors must be strengthened in order to address the remaining challenges.

## Funding

The four cohorts received funding from the following agencies: Wellcome Trust, International Development Research Center, World Health Organization, Overseas Development Administration of the United Kingdom, European Union, Brazilian National Support Program for Centers of Excellence (PRONEX), Brazilian National Council for Scientific and Tehcnological Development (CNPq), Science and Technology Department (DECIT) of the Brazilian Ministry of Health, Research Support Foundation of the State of Rio Grande do Sul (FAPERGS), Brazilian Pastorate of the Child, and Brazilian Association for Collective Health (ABRASCO).

## Pelotas Cohorts Study Group

Aluisio J D Barros,^1^ Alicia Matijasevich,^2^ Diego G Bassani,^3^ Helen Gonçalves,^1^ Joseph Murray,^1^ Luciana Tovo-Rodrigues,^1^ Maria Cecilia F Assunção,^1^ Mariangela F Silveira,^1^ Marlos Rodrigues Domingues^1^ and Pedro R C Hallal.^1^

^1^Federal University of Pelotas, Brazil, ^2^University of São Paulo, Brazil and ^3^University of Toronto, Canada.

**Conflict of interest:** None declared.

## Supplementary Material

Supplementary Tables
