# Supplementary material for: Hospital admissions in the first year of life: inequalities over three decades in a southern Brazilian city
Source: Int J Epidemiol. 2019 Mar 18;48(Suppl 1):i63–71. doi: 10.1093/ije/dyy228 (PMC6422058; doi:10.1093/ije/dyy228)
Supplement: Supplementary Tables [file dyy228_supplementary_tables.docx]

**Table S1**. Percentages of cohort children admitted to a hospital due to perinatal causes during the first year of life, according to sex, family income and maternal skin color.

| Variable | **Birth cohort year** | | | | p-value^a^ |
| --- | --- | --- | --- | --- | --- |
|  | **1982** | **1993** | **2004** | **2015** |  |
|  | **% (95% CI)** | **% (95% CI)** | **% (95% CI)** | **% (95% CI)** |  |
| **Sex** |  | p=0.704* | p=0.253* | p=0.001* |  |
| Male | – | 2.2 (1.0;3.3) | 7.4 (6.3;8.6) | 6.2 (5.2;7.3) | 0.006 |
| Female | – | 1.9 (0.8;2.9) | 6.5 (5.4;7.6) | 3.9 (3.0;4.7) | 0.664 |
| *Difference male - female* | – | 0.3 | 0.9 | 2.3 |  |
| *Ratio male/female* | – | 1.2 | 1.1 | 1.6 |  |
|  |  |  |  |  |  |
| **Family income (quintiles)** |  | p=0.483* | p=0.102* | p=0.629* |  |
| Q1 (poorest) | – | 1.8 (0.1;3.5) | 8.1 (6.2;10.0) | 5.6 (4.0;7.2) | 0.233 |
| Q2 | – | 3.3 (1.2;5.5) | 7.6 (5.8;9.5) | 5.6 (4.0;7.2) | 0.441 |
| Q3 | – | 0.8 (0.0;1.8) | 6.7 (5.0;8.5) | 4.0 (2.6;5.3) | 0.418 |
| Q4 | – | 2.3 (0.3;4.3) | 5.8 (4.2;7.4) | 4.8 (3.3;6.2) | 0.337 |
| Q5 (richest) | – | 1.5 (0.0;3.0) | 6.6 (4.9;8.4) | 5.4 (3.8;7.0) | 0.082 |
| *Slope index of inequality* | – | 0.0 (-2.8;2.8) | -2.3 (-5.2;0.5) | -0.6 (-3.0;1.8) |  |
| *Concentration index* | – | 4.4 (-15.6; 24.4) | -5.4 (-11.9; 1.1) | -0.6 (-8.6; 7.4) |  |
|  |  |  |  |  |  |
| **Maternal skin color** |  | p=0.271* | p=0.258* | p=0.606* |  |
| White | – | 2.2 (1.2;3.1) | 7.2 (6.3;8.2) | 5.0 (4.2;5.8) | 0.070 |
| Brown | – | 3.7 (0.0;8.9) | 6.6 (3.7;9.6) | 5.2 (3.3;7.1) | 0.885 |
| Black | – | 0.8 (0.0;2.0) | 6.1 (4.4;7.8) | 5.5 (3.6;7.3) | 0.017 |
| *Difference black - white* | – | -1.4 | -1.1 | 0.5 |  |
| *Ratio black/white* | – | 0.4 | 0.8 | 1.1 |  |
|  |  |  |  |  |  |

* p-values for differences within each cohort.

^a^ p-values for time trends within each category.

**Table S2**. Percentages of cohort children admitted to a hospital due to pneumonia during the first year of life, according to sex, family income and maternal skin color

| Variable | **Birth cohort year** | | | | p-value^a^ |
| --- | --- | --- | --- | --- | --- |
|  | **1982** | **1993** | **2004** | **2015** |  |
|  | **% (95% CI)** | **% (95% CI)** | **% (95% CI)** | **% (95% CI)** |  |
| **Sex** |  | p=0.513* | p=0.145* | p=0.078* |  |
| Male | – | 6.9 (4.9;9.0) | 4.9 (4.0;5.8) | 4.0 (3.2;4.9) | 0.005 |
| Female | – | 6.0 (4.2;7.9) | 3.9 (3.1;4.8) | 3.0 (2.2;3.7) | 0.001 |
| *Difference male - female* | – | 0.9 | 1.0 | 1.0 |  |
| *Ratio male/female* | – | 1.2 | 1.3 | 1.3 |  |
|  |  |  |  |  |  |
| **Family income (quintiles)** |  | p=0.002* | p<0.001* | p<0.001* |  |
| Q1 (poorest) | – | 10.2 (6.3;14.1) | 5.8 (4.1;7.4) | 4.8 (3.3;6.3) | 0.008 |
| Q2 | – | 6.3 (3.5;9.1) | 6.1 (4.4;7.8) | 4.0 (2.6;5.3) | 0.058 |
| Q3 | – | 6.2 (3.1;9.2) | 5.1 (3.5;6.6) | 4.5 (3.0;5.9) | 0.291 |
| Q4 | – | 5.7 (2.6;8.8) | 3.8 (2.5;5.1) | 2.7 (1.6;3.8) | 0.031 |
| Q5 (richest) | – | 2.8 (0.7;4.8) | 1.4 (0.6;2.3) | 1.6 (0.8;2.5) | 0.425 |
| *Slope index of inequality* | – | -5.6 (-10.6; -0.6) | -5.4 (-7.6; -3.2) | -3.8 (-5.8; -1.9) |  |
| *Concentration index* | – | -13.6 (-24.0; -2.0) | -19.4 (-26.3; -11.7) | -18.3 (-26.7; -9.9) |  |
|  |  |  |  |  |  |
| **Maternal skin color** |  | p=0.279* | p=0.083* | p=0.046* |  |
| White | – | 6.2 (4.6;7.7) | 4.0 (3.3;4.7) | 3.2 (2.5;3.8) | <0.001 |
| Brown | – | 3.8 (0.0;7.9) | 6.6 (3.7;9.6) | 4.2 (2.5;5.9) | 0.399 |
| Black | – | 8.6 (4.8;12.4) | 5.2 (3.6;6.7) | 4.6 (3.0;6.3) | 0.066 |
| *Difference black - white* | – | 2.4 | 1.2 | 1.4 |  |
| *Ratio black/white* | – | 1.4 | 1.3 | 1.4 |  |
|  |  |  |  |  |  |

* p-values for differences within each cohort.

^a^ p-values for time trends within each category.
